# C-type lectin receptors: bone remodeling and therapeutic potential

**DOI:** 10.1038/s41413-026-00567-9

**Published:** 2026-09-28

**Authors:** Marina Kleim, Aline Bozec, Manuel Weber, Bettina Groetsch

**Affiliations:** 1 Department of Medicine 3 - Rheumatology and Immunology, Friedrich-Alexander-Universität Erlangen-Nürnberg and Uniklinikum Erlangen, Erlangen, Germany; 2 Deutsches Zentrum für Immuntherapie (DZI), Friedrich-Alexander-Universität Erlangen-Nürnberg and Uniklinikum Erlangen, Erlangen, Germany; 3 Department of Oral and Cranio-Maxillofacial Surgery, Uniklinikum Erlangen, Erlangen, Germany

**Keywords:** Bone, Pathogenesis

## Abstract

Osteoimmunology explores the interplay between the skeletal system and the immune system. It focuses on the identification of shared signaling pathways and the mechanisms of mutual regulation. Bone homeostasis depends on the balance between the activity of bone-forming osteoblasts and bone-resorbing osteoclasts. Interestingly, the crosstalk between osteoclasts, osteoblasts, osteocytes, and immune cells not only shapes bone remodeling but also influences immune responses. A central pathway in osteoclast differentiation is the receptor activator of nuclear factor-κB ligand (RANKL) signaling, which induces NFATc1, the master transcription factor of osteoclastogenesis. Recent evidence shows that immunoreceptor tyrosine-based activation motif (ITAM)-containing co-receptors enhance RANKL-induced signaling, underscoring their essential role in osteoclast activation and differentiation. Increasing attention has been directed toward C-type lectin receptors (CLRs), such as Dectin-1, Mincle, and DC-SIGN. These receptors have historically been recognized for their functions in antifungal and antibacterial immunity. However, their expression on both immune cells and osteoclasts links CLR signaling to osteoclastogenesis, as CLR engagement signals through ITAM pathways, contributing to NFAT activation. Therefore, CLRs could function as co-receptors for RANKL in human osteoclast biology, although the underlying mechanisms remain poorly defined. This review synthesizes current knowledge on CLR signaling and its role in osteoclastogenesis. It examines how CLR-mediated pathways influence bone resorption and evaluates their therapeutic potential. Finally, the review discusses the challenges of translating these insights into clinical applications and highlights emerging strategies for targeting CLRs in bone-related pathologies.

## Introduction

Bone remodeling is tightly linked to immune regulation and is essential for maintaining skeletal integrity and tissue homeostasis. Chronic inflammation disrupts the balance between osteoclasts and osteoblasts, leading to increased bone resorption and pathological bone loss in diseases such as rheumatoid arthritis, periodontitis, osteoporosis, and osteomyelitis.^1–3^

In this context, pattern-recognition receptors (PRRs), particularly C-type lectin receptors (CLRs), have emerged as important regulators at the interface of immunity and bone metabolism. CLRs recognize microbial and endogenous ligands and modulate inflammatory responses, tissue repair, and osteoclast differentiation.^4–6^ Several myeloid CLRs activate signaling pathways that intersect with classical osteoclastogenic pathways, thereby influencing inflammatory bone remodeling.^7^

Of particular interest are the ITAM- and hemITAM-associated CLRs Dectin-1 (CLEC7A) and Mincle (CLEC4E), as well as the immunomodulatory receptor DC-SIGN (CD209). These receptors activate intracellular signaling pathways involving Syk kinase, CARD9, NF-κB, MAPK, and NFATc1, which closely interact with RANKL-mediated osteoclastogenic signaling.^8,9^ Experimental evidence suggests that CLR signaling can either enhance or suppress osteoclastogenesis depending on ligand structure, inflammatory context, receptor crosstalk, and the cellular microenvironment. In addition, recent studies indicate that CLR-mediated trained immunity in myeloid progenitors may establish long-lasting pro-osteoclastogenic programs, linking chronic inflammation and microbial exposure to sustained bone destruction.^10–17^

Persistent activation of Dectin-1, Mincle, and DC-SIGN during chronic inflammatory conditions promotes cytokine production, enhances osteoclast activity, and contributes to pathological bone loss.^18–23^ Together, these findings identify CLRs as central regulators of osteoimmune interactions and promising therapeutic targets in inflammatory bone diseases. However, the context-dependent nature and complexity of CLR signaling continue to limit their translational application and warrant further investigation.

This review summarizes the current knowledge of the structure, classification, and signaling mechanisms of myeloid CLRs, focusing particularly on Dectin-1, Mincle, and DC-SIGN. We also discuss their emerging roles in osteoclast differentiation, bone remodeling, and inflammatory bone diseases, such as rheumatoid arthritis (RA), osteoporosis, and periodontitis. Finally, we discuss the therapeutic potential of targeting CLR-mediated pathways.

## C-type lectin receptors

The innate immune system relies on a repertoire of PRRs to sense invading pathogens and tissue damage.^24,25^ Among these, the CLRs represent one of the most diverse and functionally versatile families. Defined by their C-type lectin-like domain (CTLD), CLRs recognize a broad spectrum of pathogen-associated molecular patterns (PAMPs) as well as endogenous danger-associated molecular patterns (DAMPs).^4–6^ Through these interactions, they contribute to the initiation of innate immune responses and the orchestration of adaptive immunity.

### CLRs classification and structure

Structurally, CLRs possess a characteristic carbohydrate recognition domain (CRD), a compact module formed by two protein loops stabilized by disulfide bridges. This architecture underlies their ability to engage with glycans, often in a calcium-dependent manner. While glycan recognition is considered a hallmark of CLRs, accumulating evidence indicates that some family members also bind non-carbohydrate ligands, including cholesterol and uric acid crystals, highlighting their adaptability in immune surveillance.^4,26^

The CLR superfamily is the largest lectin receptor family in animals, encompassing 17 phylogenetically defined groups (I–XVII). C-type lectin domain-containing (CLEC) proteins constitute a family of receptors characterized by the presence of one or more CTLDs, many of which play important roles in immune recognition. Genes encoding these proteins are commonly designated using the CLEC nomenclature (e.g., CLEC4E, CLEC7A). Within the context of innate immunity, particular attention has been directed toward myeloid CLRs, which are predominantly expressed by macrophages and dendritic cells. These receptors, which fall primarily within groups II, V, and VI, function as key modulators of host defense and immune homeostasis. They operate either independently or in synergy with other PRRs to regulate pathogen uptake, antigen processing, cytokine production, and inflammatory signaling.^5,6,26^

**Fig. 1:**
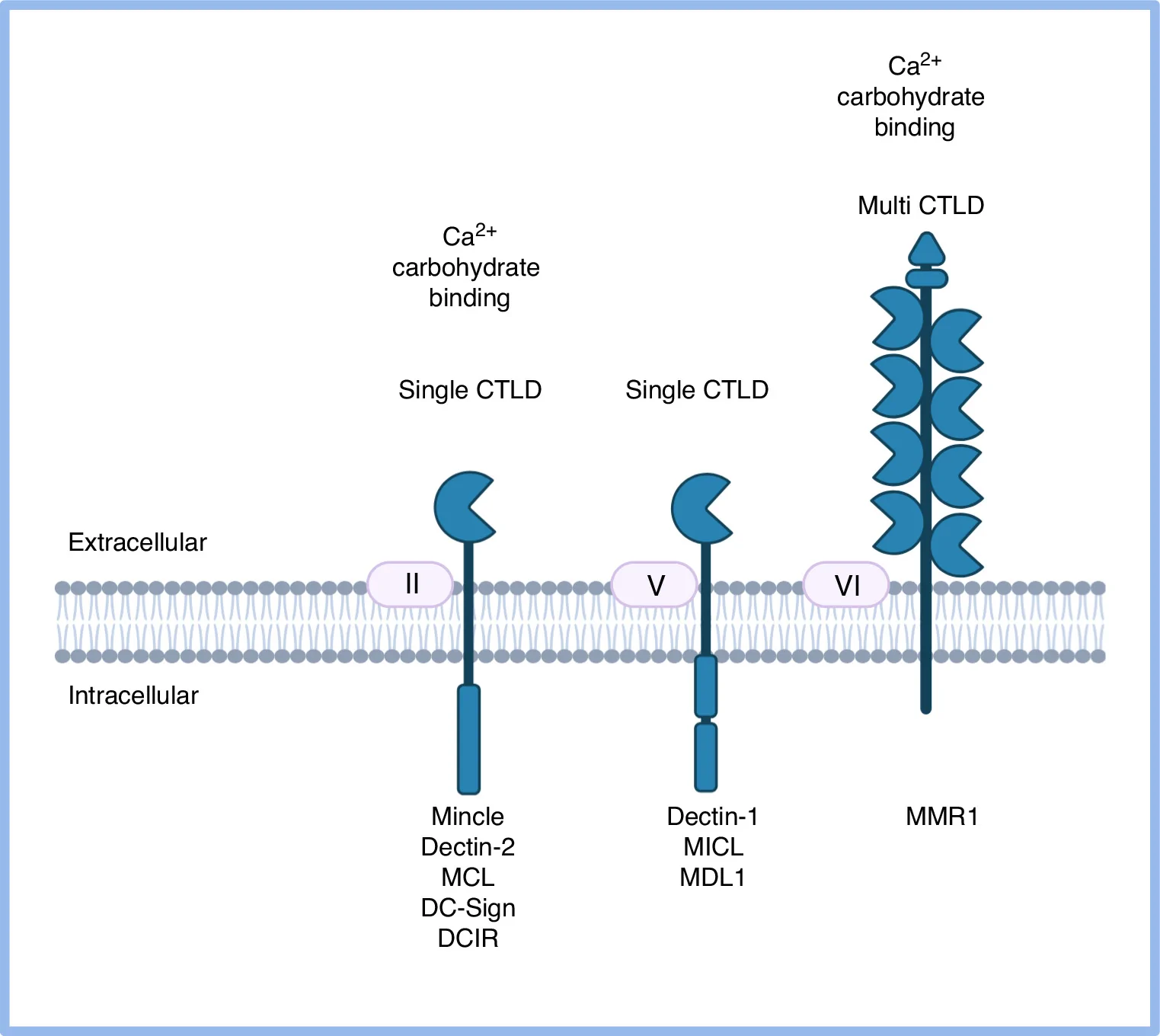
Classification and structure of myeloid C-type lectin receptors. The three myeloid CLR-containing groups (II, V, VI) are highlighted. Characteristic structural features of the three groups are shown, and affiliated members of the groups are listed. CTLD (C-type lectin-like domain)

Groups II and VI contain canonical C-type lectin domains with conserved residues required for Ca²⁺-dependent carbohydrate binding. In contrast, group V members, including Dectin-1 (CLEC7A), lack some of these canonical Ca²⁺-coordinating residues and therefore do not mediate classical mannose-type recognition. However, they retain carbohydrate-binding capacity and have evolved to recognize β-glucans in a Ca²⁺-independent manner. Groups II and V are restricted to a single CTLD, in contrast to the multi-CTLD arrangement of group VI. Functionally, group II CLRs facilitate pathogen binding and uptake, group V CLRs predominantly engage in signaling roles, and group VI CLRs specialize in antigen capture and processing (Fig. 1).

#### Group II CLRs

Group II CLRs are type II transmembrane proteins characterized by a short cytoplasmic tail, a transmembrane domain, an extracellular stalk region, and a single CTLD. The length of the stalk is variable and influences receptor oligomerization. Members of this group, such as DC-SIGN (CLEC4L), DCIR (CLEC4A), Mincle (CLEC4E), and Dectin-2 (CLEC6A), contain CRDs with canonical Ca²⁺- and carbohydrate-binding motifs.^27^ They play pivotal roles in pathogen recognition and are strongly implicated in shaping adaptive immunity through their capacity for antigen capture and presentation.^4,28^ For example, DC-SIGN binds the oligomannose gp120 of the HIV-1 virus, facilitating its internalization. It then presents the internalized ligands to CD4^+^ T cells via MHC II. Meanwhile, DCIR acts as an attachment factor, capturing HIV-1.^29–31^

#### Group V CLRs

Group V CLRs also adopt a type II transmembrane organization; however, their single CTLD often lacks some of the conserved residues required for classical Ca²⁺-dependent carbohydrate binding. Although they lack the canonical Ca²⁺-dependent carbohydrate-binding features, this does not imply a complete loss of carbohydrate recognition. For example, members such as Dectin-1 can still bind carbohydrates, including β-glucans, through non-classical, Ca²⁺-independent mechanisms. Members such as Dectin-1 (CLEC7A), MDL-1 (CLEC5A), and MICL (CLEC12A) are crucial immune regulators. They contribute to intracellular signaling pathways that modulate inflammation and host defense.^4,28^ Dectin-1, for example, is well known for its role in antifungal immunity such as *Aspergillus*, *Candida* and *Saccharomyces*, operating through recognition of β-glucans.^24,32^

#### Group VI CLRs

Group VI CLRs differ fundamentally from groups II and V, since they are type I transmembrane receptors containing multiple extracellular CTLDs (8–10). In addition, they often possess an N-terminal ricin-like domain and a fibronectin type 2 domain. The macrophage mannose receptor (MMR) and DEC-205 exemplify this subgroup. Their structural complexity reflects their functional specialization, as these receptors are primarily involved in endocytosis and antigen presentation rather than direct immune signaling.^4,33^ Therefore, MMR, for example, mediates the clearance of *Leishmania* and *Klebsiella pneumoniae*.^34,35^

Taken together, these three groups of myeloid CLRs reveal both structural convergence and divergence.

### CLRs transmembrane and intracellular domains

The transmembrane domain not only anchors CLRs within the plasma membrane but also connects extracellular ligand recognition with intracellular signaling. Typically, these hydrophobic regions span the membrane with ~25 amino acids. Upon ligand binding, conformational changes within the transmembrane helix can propagate structural rearrangements toward the cytoplasmic signaling motifs.^36^ An illustrative example is the macrophage inducible C-type lectin (Mincle), which harbors a conserved positively charged arginine within its transmembrane domain. This residue mediates association with the negatively charged immunoreceptor Fc receptor γ (FcRγ) chain, which bears an ITAM. Mutation of this arginine to a neutral residue, such as isoleucine, abrogates FcRγ binding, highlighting the essential contribution of the transmembrane domain to CLR signaling.^37^

#### Functional classes of CLR intracellular domains

The cytoplasmic domains of myeloid CLRs dictate signaling outcomes. Based on the presence and type of signaling motifs, four principal classes have been defined^4,5,38^ (summarized in Table 1).

- *ITAM-coupled CLRs*: ITAM-bearing motifs are characterized by tandem YxxL/I repeats (Y = tyrosine, x = any residue, L/I = leucine or isoleucine). Many CLRs lack intrinsic ITAMs but signal via association with adapter proteins such as FcRγ or DNAX-activating protein 12 (DAP12), both of which harbor canonical ITAMs. Ligand engagement leads to ITAM phosphorylation, recruitment of spleen tyrosine kinase (SYK) via its SH2 domains, and subsequent activation through autophosphorylation. Representative ITAM-coupled CLRs include Mincle (CLEC4E), Dectin-2 (CLEC6A), DCAR (CLEC4B) and MDL-1 (CLEC5A).^39–41^
- *hemITAM-bearing CLRs*: a subset of CLRs contains a single YxxL/I motif within their cytoplasmic tail, termed a hemITAM. Although less extensive than a full ITAM, the hemITAM is sufficient for SYK recruitment and activation following ligand binding. Well-characterized hemITAM CLRs include Dectin-1 (CLEC7A), CLEC-2, DNGR-1 (CLEC9A), and SIGNR3. These receptors mediate robust immune activation by bridging pathogen recognition to SYK-dependent pathways.^42,43^
- *ITIM-bearing CLRs*: in contrast to activating receptors, ITIM-bearing CLRs function as negative regulators. Their cytoplasmic ITIM motifs (I/VxYxxL) recruit tyrosine phosphatases such as SHP-1 and SHP-2, thereby dampening activation signals. These inhibitory CLRs often modulate responses when engaged simultaneously with activating receptors, fine-tuning immune outcomes rather than initiating signaling alone. Examples include human DCIR (CLEC4A), murine orthologues mDCIR1 (CLEC4A2) and mDCIR2 (CLEC4A4), as well as CLEC12A (MICL).^4,7,9^
- *CLRs without canonical ITAM/ITIM motifs*: a fourth group of myeloid CLRs lacks obvious signaling motifs, but they play important roles in antigen uptake and immune modulation. These receptors generally mediate endocytosis, antigen processing, and presentation to T cells rather than direct immune activation. While some, such as DC-SIGN, can influence signaling indirectly through defined pathways, the underlying mechanisms remain poorly characterized for many. Members include MMR, DEC-205, DC-SIGN, Langerin, and macrophage galactose-type lectin (MGL).^44–46^

**Table 1:** Intracellular domains of myeloid C-type lectin receptors

CLR class	Motif/Adapter	Mechanism of signaling	Representative members	Functional outcome
ITAM-coupled CLRs	ITAM in adapter proteins (FcRγ, DAP12)	Ligand engagement → ITAM phosphorylation → SYK recruitment	Mincle (CLEC4E), Dectin-2 (CLEC6A), DCAR (CLEC4B), MDL-1 (CLEC5A),	Strong immune activation, cytokine production
hemITAM-bearing CLRs	Single YxxL/I motif in cytoplasmic tail	hemITAM phosphorylation → SYK recruitment/activation	Dectin-1 (CLEC7A), CLEC-2, DNGR-1 (CLEC9A),	SYK-dependent immune activation, phagocytosis, cytokine induction
ITIM bearing CLRs	ITIM motif (I/VxYxxL)	Recruitment of SHP-1/2 phosphatases	DCIR (CLEC4A), mDCIR1 (CLEC4A2), mDCIR2 (CLEC4A4), MICL (CLEC12A), CLEC12B	Negative regulation of immune activation, immune tolerance
Motif-independent CLRs	Lack canonical ITAM/ITIM motifs	Mediate endocytosis; in some cases, modulate signaling via undefined pathways	Mannose receptor (MMR), DEC-205, DC-SIGN, Langerin, MGL (hMGL, mMGL1/2), LOX-1	Antigen uptake, processing, presentation; modulation of other signaling cascades

### CLRs signaling pathways in macrophages and dendritic cells

#### ITAM and hemITAM-dependent activating CLRs

**Fig. 2:**
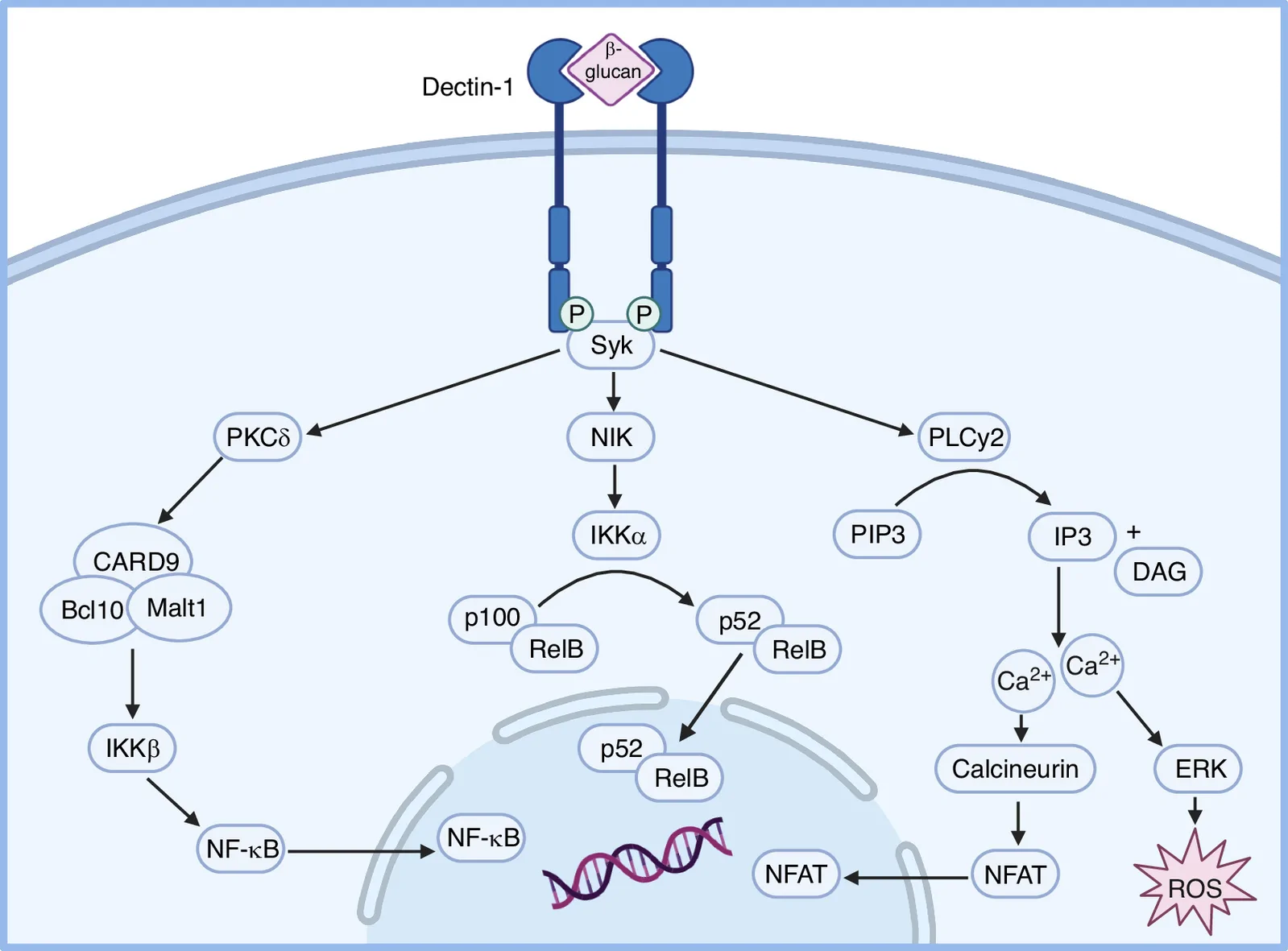
Dectin-1 (Clec7a) hemITAM signaling pathways. Upon ligand binding, e.g., β-glucans, Dectin-1 receptor cluster together. Their ITAM-like motif gets phosphorylated and recruits Syk. Syk activates the kinases PKCδ, NIK, and PLCγ2. PKCδ induces the CARD8-Bcl10-Malt1 complex, resulting in the nuclear translocation of NF-κB. NIK causes the degradation of p100 and the formation of p52/RelB dimers. Dectin-1 signaling also promotes the generation of ROS and nuclear translocation of NFAT by activating PLCγ2 and resulting in calcium influx

A substantial subset of CLRs signal via ITAM or hemITAM motifs that recruit and activate Syk kinase following ligand engagement. Prototypical examples include Mincle (CLEC4E) and Dectin-1 (CLEC7A), which recognize microbial glycolipids or fungal β-glucans and initiate Syk-dependent signaling cascades (Fig. 2). The activation of Syk converges on the activation of the CARD9–Bcl10–Malt1 complex, canonical, and non-canonical NF-κB, MAPK, and NFAT signaling, resulting in robust production of pro-inflammatory cytokines (e.g., TNF-α, IL-6, IL-1β, CXCL1/2), reactive oxygen species, and inflammasome activation (Fig. 2).^47–49^ In addition to canonical signaling, CLR engagement activates non-canonical NF-κB pathways through NF-κB–inducing kinase (NIK) and IKKα-mediated processing of p100 into p52, resulting in transcriptionally active p52–RelB dimers.^50^ In parallel, Raf-1 kinase modulates NF-κB output by promoting p65–RelB dimerization, thereby fine-tuning inflammatory gene expression. CLR-induced activation of phospholipase Cγ2 (PLCγ2) and subsequent calcium influx further diversifies signaling by triggering NFAT activation, driving the expression of cytokines such as IL-2 and IL-10. Moreover, PKCδ- and ERK-dependent reactive oxygen species (ROS) production plays a central role in NLRP3 inflammasome activation and IL-1β maturation.^51–53^

These integrated pathways enable CLRs to orchestrate a broad range of immune effector functions, from cytokine release to inflammasome activation and T cell polarization. The strength and kinetics of ITAM/hemITAM signaling appear to influence distinct cellular outcomes, though the precise mechanisms underlying differential responses among CLRs such as CLEC4E and CLEC6A remain incompletely understood.

#### Alternative CLR signaling pathways

Not all CLRs rely on Syk-mediated ITAM signaling. DC-SIGN (CD209), for example, engages a Raf-1–dependent pathway via a signalosome containing LSP1, KSR1, and CNK. This pathway modifies NF-κB transcriptional activity rather than directly inducing cytokine production. DC-SIGN thereby functions primarily as a signaling modulator or an endocytic receptor involved in antigen processing and presentation, rather than as a classical activator of dendritic cells.^54^ Interestingly, DC-SIGN and other non-ITAM/ITIM CLRs can induce distinct signaling outcomes depending on the ligand encountered, reflecting their context-dependent plasticity. Crosstalk between CLRs and other PRRs adds another layer of complexity. For instance, Dectin-1 and DC-SIGN can cooperate with Toll-like receptors (TLRs) to amplify antimicrobial immunity, whereas interactions between CLEC2D and TLR2 have been shown to suppress protective responses.^39,55^ These examples highlight how receptor co-engagement and spatial proximity fine-tune immune outcomes.

#### ITIM-containing CLRs and immune suppression

In contrast to activating CLRs, a subset of myeloid CLRs harbor immunoreceptor tyrosine-based inhibitory motifs (ITIMs). These receptors recruit phosphatases such as SHP-1 and SHP-2 to counterbalance activator signals and maintain immune homeostasis. Examples include human DCIR (CLEC4A), murine DCIR homologs (Clec4a2, Clec4a4), CLEC12A, CLEC12B, and Ly49Q.^5,45^ Activation of hDCIR on dendritic cells inhibits TLR8-mediated IL-12 and TNF-α production, as well as TLR9-induced IFN-α secretion.^56^ In addition to suppressing inflammatory responses to microbial ligands, ITIM-bearing CLRs play essential roles in sensing endogenous ligands and regulating inflammation in the context of autoimmune disease or chronic infection (e.g., Plasmodium, Mycobacterium).^7,57^

While CLRs are primarily recognized for their roles in pathogen recognition and immune modulation, emerging evidence highlights their expression on bone-resident cells, including osteoclasts and osteoblasts. Through ITAM-mediated crosstalk with pathways like RANKL signaling, CLRs fine-tune osteoclastogenesis and bone resorption, positioning them as key players at the intersection of immunity and skeletal homeostasis.

## Bone remodeling

Bone remodeling is a highly dynamic and lifelong process that sustains skeletal integrity and mineral homeostasis. This continuous turnover is carried out within basic multicellular units, where a precise sequence of bone resorption and bone formation ensures structural renewal and adaptation to mechanical and metabolic demands. The process depends on the coordinated actions of three major cell types: osteoclasts, osteoblasts, and osteocytes.^1,2^

Osteoclasts, derived from hematopoietic precursors of the monocyte–macrophage lineage, are the primary bone-resorbing cells. Through the secretion of protons and proteolytic enzymes, they dissolve the mineralized matrix and degrade the organic bone scaffold. Their differentiation and activation are tightly controlled by the RANK/RANKL/OPG signaling axis and co-stimulatory ITAM adapter pathways.^58^

Unlike osteoclasts, osteoblasts, and their terminally differentiated form, osteocytes derive from multipotent mesenchymal progenitors. Osteoblasts are located on both the inner (endosteal) and outer (periosteal) bone surfaces, where their main function is the production and secretion of type I collagen and alkaline phosphatase to form the bone matrix. This matrix is subsequently mineralized extracellularly.^59,60^ This process helps maintain bone’s strength and density. In case of decreased bone formation, bones become weak and more susceptible to fractures. Furthermore, osteoblasts and osteocytes also regulate bone resorption by osteoclasts via the receptor activator of nuclear factor κB ligand (RANKL). Following bone formation, osteoblasts undergo distinct fates: apoptosis, conversion into bone-lining cells, or entrapment within the matrix to become osteocytes. Osteocytes are embedded within the mineralized bone matrix and constitute the most abundant cell population in bone. Through their extensive lacuno–canalicular network, osteocytes serve as the primary mechanosensors of bone and play a central role in the regulation of bone remodeling.^61^ They produce key regulatory factors, including RANKL and sclerostin, a potent inhibitor of osteoblast activity, thereby coordinating mechanical loading, endocrine signals, and cellular interactions to maintain skeletal homeostasis.^62,63^

**Fig. 3:**
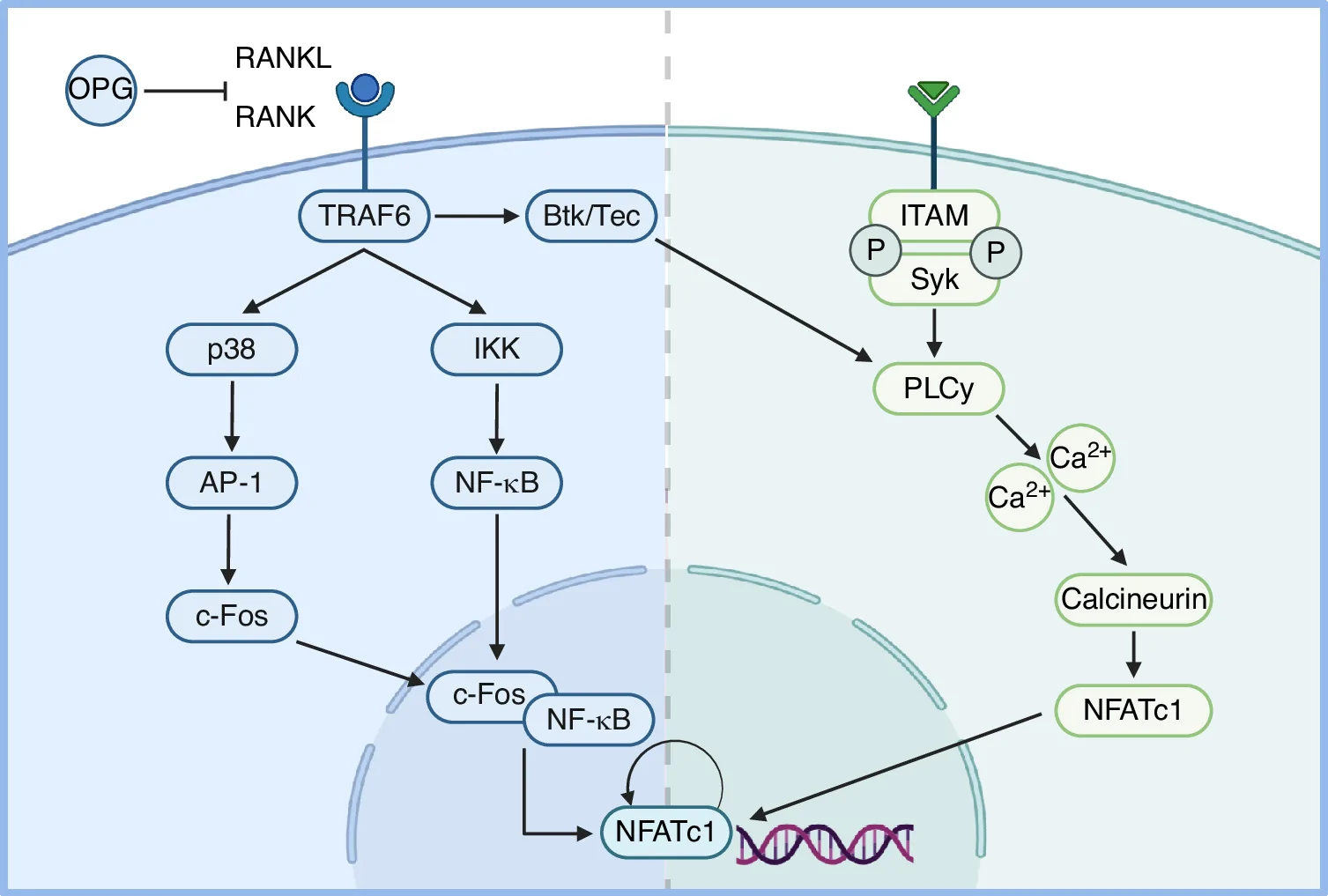
RANK signaling in osteoclasts after RANKL binding. TRAF6 is recruited by RANK-RANKL, which activates the canonical NF-κB pathway as well as AP-1, c-Fos. IKK releases NF-κB, allowing its translocation into the nucleus. Additionally, TRAF6 induces c-Fos, which in turn activates, together with NF-κB, the expression of NFATc1 (blue). Co-stimulation of immunoreceptors via ITAM motifs leads to the release of intracellular calcium with the help of BTK and Tec kinases, together with PLCγ2. This activates calcineurin, which facilitates the translocation of NFATc1 into the nucleus (green)

In inflammatory diseases such as rheumatoid arthritis, immune cell–derived RANKL and pro-inflammatory cytokines disrupt bone homeostasis by enhancing osteoclastogenesis and driving localized bone destruction.^64,65^ Central to this process is the RANK/RANKL/OPG axis, which governs osteoclast differentiation and activation. RANKL, expressed by osteoblasts, osteocytes, stromal cells, and activated T and B lymphocytes, engages with RANK on osteoclast precursors, leading to TRAF6 recruitment and activation of canonical NF-κB and AP-1 transcription factors, including c-Fos and Fra-1.^66–69^ These pathways converge to induce NFATc1, the master regulator of osteoclastogenesis (Fig. 3).^70^

While osteoprotegerin (OPG) limits this signaling by binding and neutralizing RANKL, inflammatory cytokines exert additional, direct effects on osteoclast lineage cells. Pro-inflammatory mediators such as TNF-α, IL-1, IL-6, and IL-17 directly enhance osteoclast survival and resorptive function by activating NF-κB, MAPK, and AP-1 pathways and synergizing with RANK signaling, whereas IL-10, TGF-β, and IFN-γ suppress osteoclastogenesis by inhibiting these pathways.^71^ Persistent cytokine-driven osteoclast activation contributes to pathological bone loss, including osteoporosis, which is particularly prominent in postmenopausal women due to estrogen deficiency–induced shifts in osteoclast activity and lifespan.^72–74^

Beyond RANKL signaling, ITAM adapter pathways provide essential co-stimulatory signals for osteoclast differentiation.^75^ The adapters DAP12 and FcRγ, associated with receptors such as TREM-2 and OSCAR, recruit Syk kinase upon activation and induce calcium-dependent NFATc1 signaling.^75–77^ These ITAM-mediated pathways integrate with RANKL-driven NF-κB and MAPK signaling to ensure full osteoclast maturation, highlighting a cooperative immune–bone signaling network whose dysregulation underlies both defective bone remodeling and inflammatory bone destruction (Fig. 3).^78^

Inflammation adds an additional layer of regulations. Acute inflammation, as observed during fracture healing, promotes the recruitment of osteoclasts to remove damaged bone and mobilizes mesenchymal stem cells for osteoblast differentiation. In contrast, chronic inflammation, characteristic of autoimmune diseases such as rheumatoid arthritis and periodontitis, drives sustained osteoclastogenesis, suppresses osteoblast function, and results in pathological local and systemic bone loss.^3^ The persistent production of RANKL and pro-inflammatory cytokines, together with aberrant ITAM signaling, contributes to this uncoupling of bone formation and resorption.^18^

## CLRs in bone biology

Emerging evidence increasingly highlights the critical role of myeloid ITAM-bearing CLRs in regulating osteoclast differentiation, function, and overall bone homeostasis. Through their ITAMs, myeloid CLRs engage signaling pathways that intersect with key osteoclastogenic mechanisms, thereby influencing bone resorption and remodeling. In the following section, we summarize current insights into how ITAM-associated CLRs modulate osteoclast development and activity, discuss their crosstalk with other immune and bone-regulatory pathways, and explore their emerging relevance in physiological and pathological bone remodeling.

### Dectin-1 (CLEC7A)

#### Dectin-1 structure, ligand recognition, and signaling

**Fig. 4:**
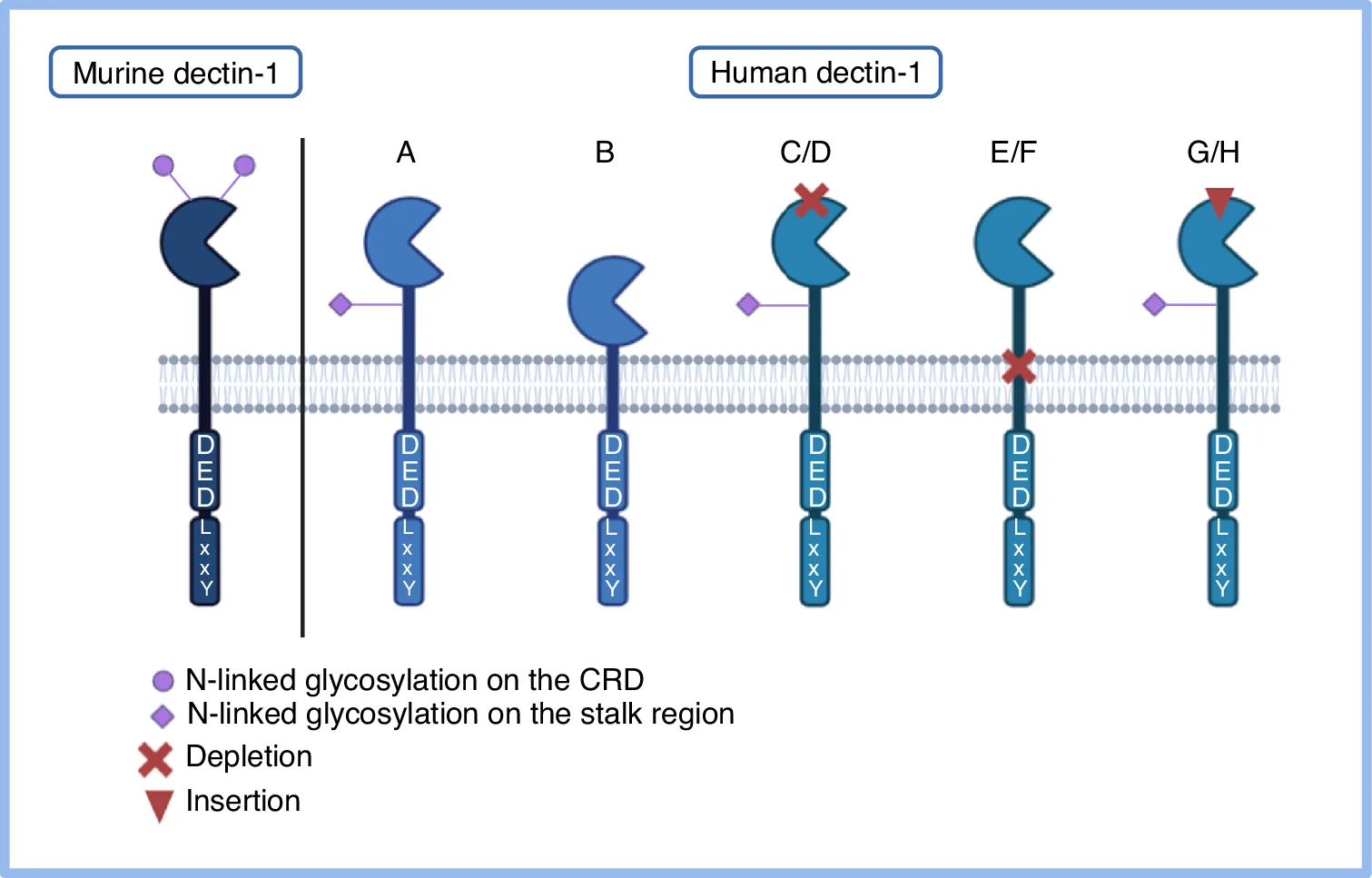
Overview of the human and murine Dectin-1 isoforms. Dectin-1 has an extracellular carbohydrate recognition domain (CRD) linked to the transmembrane region by a stalk. Intracellular Dectin-1 contains a tri-acid motif (DED) and the ITAM-like motif (YxxL). Murine Dectin-1 undergoes N-linked glycosylation on the CRD (pink circle). The two primary human isoforms A and B differ in their stalk region. Isoform A has an N-linked glycosylation on the stalk region (pink diamond), while isoform B lacks the stalk region. Isoforms C and D have deletions in the CRD, isoforms E and F have deletions in the transmembrane region, and isoforms G and H have an insertion in the CRD

Dectin-1 is a group V transmembrane CLR composed of a single Ca²⁺-independent CRD-like domain linked to a transmembrane region via a stalk and terminating in a cytoplasmic tail containing an ITAM-like motif for downstream signaling (Fig. 4).^4^ A tri-acid (DED) motif proximal to the membrane is essential for phagocytosis,^24^ and specific CRD residues (Trp221, His223) are required for β-glucan binding.^79^ The human CLEC7A gene undergoes alternative splicing, generating several isoforms (A–H), though only isoforms A and B are functionally significant.^80,81^ Compared with murine Dectin-1, the human receptor is smaller, possessing a single N-linked glycosylation site.^81,82^ Dectin-1 is expressed on neutrophils, eosinophils, monocytes, and macrophages, but could also be detected on B and T lymphocytes.^83^

Functionally, Dectin-1 recognizes carbohydrate polymers containing β(1→3)- and β(1→6)-linked glucans, which are major components of fungal cell walls and are also present in certain plants and bacteria. Through this recognition, Dectin-1 detects pathogens such as Candida, Pneumocystis, and Saccharomyces, underscoring its central role in antifungal immunity.^79,84^ Beyond its interaction with microbial β-glucans, Dectin-1 also binds endogenous ligands, including galectin-9 and annexins (Table 2),^85–88^ highlighting its broader involvement in immunomodulation and tissue homeostasis beyond pathogen recognition.

**Table 2:** Ligands for Dectin-1 and their pathology

Ligands	Pathology	Reference
Annexins	Autoimmune diseases and aging	^85,88^
β-glucans	Microbial infections	^24,79,130^
Galactosyated immunoglobulins	Anti-inflammatory	^131^
Galectin-9	Autoimmune diseases and Cancer	^86,87^
*Mycobacterium tuberculosis*	Mycobacterial infection	^53,132^
Vimentin	Atherosclerosis and obesity	^133^

#### Dectin-1 in osteoclastogenesis and bone remodeling

**Fig. 5:**
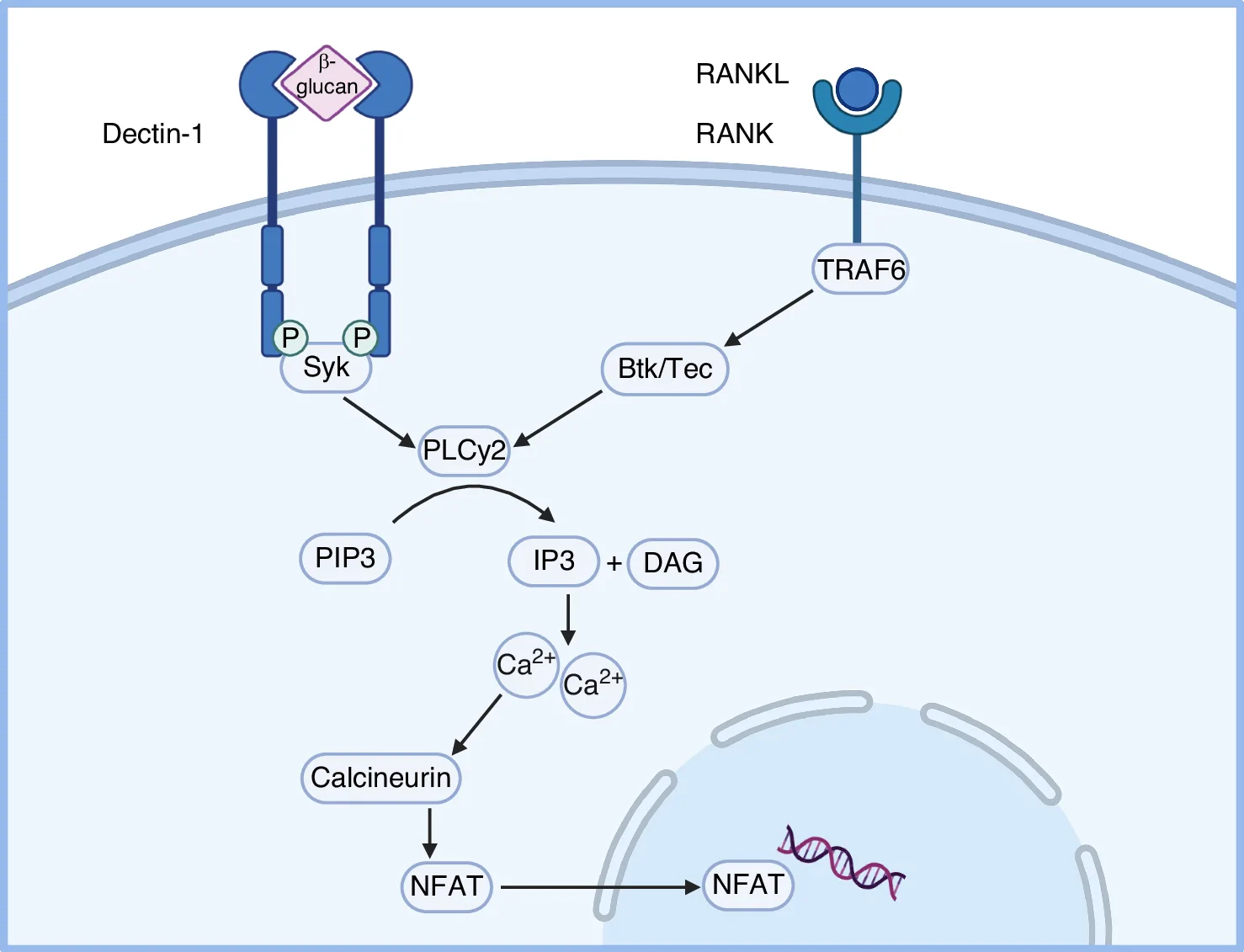
Integration of RANK and Dectin-1 signaling in osteoclasts. Dectin-1’s ITAM-like motif induces Syk signaling, which in turn induces PLCγ2 and a resulting calcium influx. PLCγ2 is activated through bridging the RANK and ITAM downstream signaling by Btk and Tec. This activates calcineurin, allowing NFATc1 (NFAT) to translocate into the nucleus. Thereby, RANK signaling and co-stimulation of immune receptors with an ITAM motif activate NFAT to regulate osteoclastogenesis

Dectin-1 is expressed on myeloid cells, including murine and human osteoclasts.^17^ Madel et al. demonstrated distinct Dectin-1 expression patterns in osteoclast subtypes: inflammatory osteoclasts, which promote TNFα-producing CD4⁺ T cells, express higher Dectin-1 levels than tolerogenic osteoclasts, which induce regulatory T cells.^15^ These findings implicate Dectin-1 in the regulation of osteoclastogenesis, particularly under inflammatory conditions. Mechanistically, both RANKL and Dectin-1 signaling converge on PLCγ2 activation, leading to the induction of NFATc1. Since RANKL signaling depends on Syk-mediated ITAM or ITAM-like motifs to activate PLCγ2, Dectin-1, through its own ITAM-like motif, may contribute to osteoclastogenesis via shared signaling intermediates (Fig. 5).^70^ Although this has been demonstrated primarily in murine models, the structural and functional similarity between murine and human Dectin-1 suggests a conserved mechanism that warrants further exploration in human systems.

Dectin-1 activation influences osteoclast biology not only by directly regulating osteoclast differentiation and activity, but also by inducing trained immunity programs in myeloid precursors that later give rise to osteoclasts.

β-Glucans, the canonical ligands of Dectin-1 present in fungi, plants, and bacteria, modulate osteoclastogenesis both through direct engagement of Dectin-1 on osteoclast precursors and by imprinting a sustained pro- or anti-osteoclastogenic bias within the myeloid compartment.^89,90^ In this context, curdlan, a linear β-(1,3)-D-glucan and selective Dectin-1 agonist, illustrates how Dectin-1-dependent training can reprogram signaling pathways such as Syk–CARD9 and downstream transcriptional networks, thereby shaping subsequent RANKL-driven osteoclast differentiation and function even after the initial stimulus has resolved.^17,91–93^

Dectin-1 activation further induces the transcription of MafB and Bcl6, both negative regulators of NFATc1. In addition, Dectin-1 enhances IL-33 expression in osteoclast precursors, which in turn, reinforces MafB expression and inhibits osteoclast differentiation, although its levels following curdlan stimulation may be insufficient for full suppression.^93^ Consistent with these findings, β-glucans from baker’s yeast (*Saccharomyces cerevisiae*) inhibit RANKL-induced osteoclastogenesis by downregulating NFATc1, c-Fos, and NF-κB nuclear translocation, while promoting Syk degradation through proteasomal and autophagic pathways.^94^ These results collectively suggest that Dectin-1 activation exerts an inhibitory effect on osteoclastogenesis via suppression of NFATc1 signaling in vitro. However, the molecular heterogeneity of β-glucans strongly influences their bioactivity. Linear β-glucans such as curdlan exhibit greater inhibitory potential than branched variants derived from black yeast, reflecting a ligand-specific effect. While most β-glucans attenuate osteoclast formation and bone resorption, others, such as zymosan from *S. cerevisiae*, can promote inflammatory bone loss and osteoclast activation.^11^ This duality underscores a context-dependent role for Dectin-1 signaling, likely shaped by ligand structure, receptor crosstalk, and the presence of endogenous ligands. Notably, Dectin-1 engagement not only induces IL-33 but also increases TNF-α and IL-1β production, both of which enhance osteoclast differentiation.^14,16^ Furthermore, curdlan-mediated Dectin-1 activation appears to selectively inhibit inflammatory, but not tolerogenic, osteoclasts, suggesting subtype-specific effects.^15,17^ Therefore, clarifying Dectin-1 cell- and species-specific mechanisms, particularly in human systems, will be critical for translating these insights into therapeutic strategies targeting osteoclast-related bone diseases.

### Mincle (CLEC4E)

#### Mincle structure, ligand recognition, and signaling

**Fig. 6:**
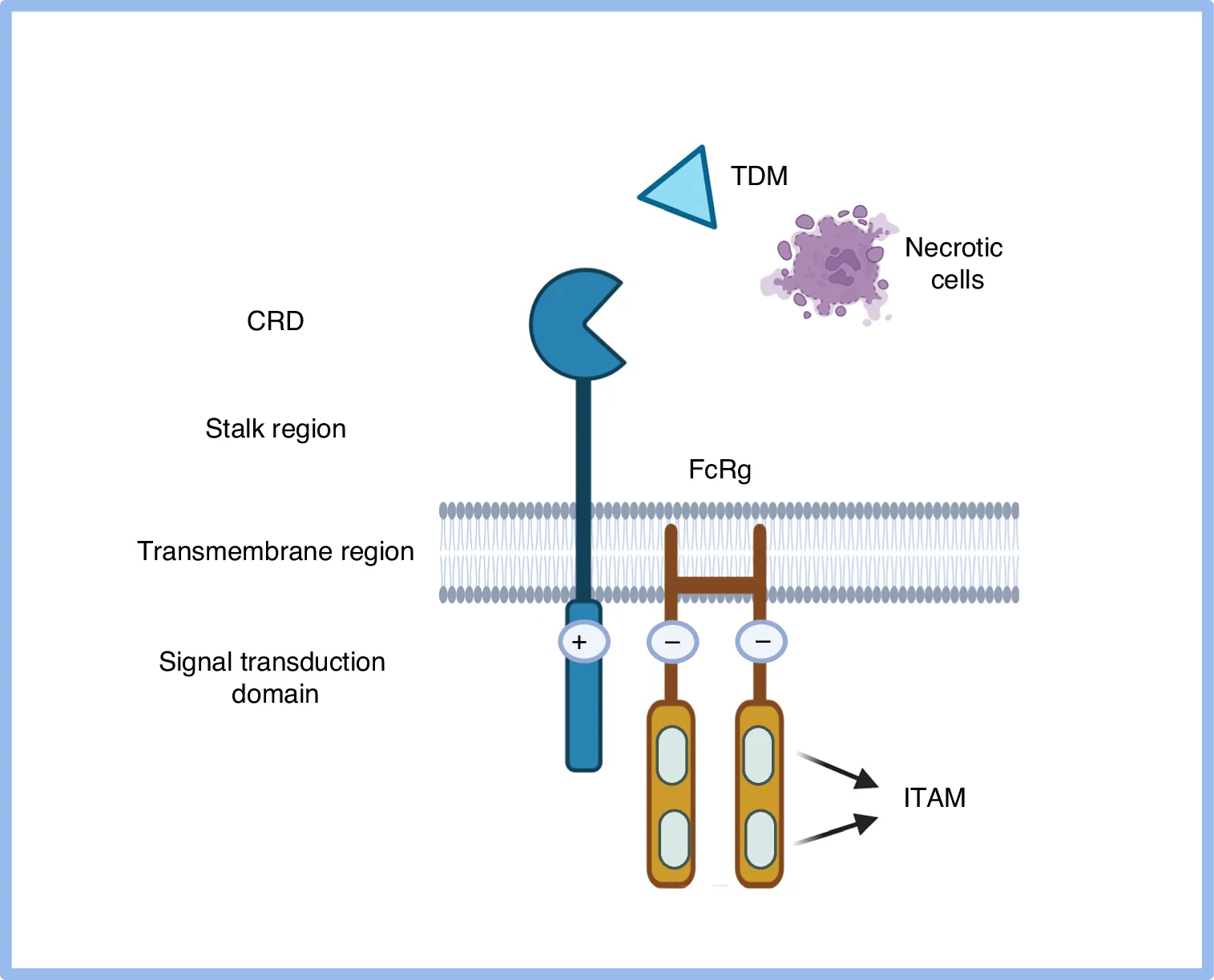
Mincle structure and intracellular binding motif. Mincle consists of a single extracellular CRD, a variable-length stalk region, a transmembrane region, and a short cytoplasmic domain free of tyrosine residues. Among them, CRD contains conserved residues that coordinate two Ca^2+^, responsible for binding to carbohydrates. Moreover, a positively charged arginine residue exists in the transmembrane region, which drives Mincle to couple with FcRγ and transmits the activation signal through ITAM in FcRγ

Macrophage-inducible C-type lectin (Mincle, CLEC4E) is a type II transmembrane CLR that functions as a key immunostimulatory PRR sensing both pathogen- and damage-associated molecular patterns (PAMPs and DAMPs). Originally identified as a receptor for the mycobacterial cord factor trehalose-6,6′-dimycolate (TDM), Mincle has emerged as an important modulator of immune responses, inflammation, and tissue homeostasis.^10,95^ Structurally, Mincle belongs to the group II CLRs and contains a single extracellular CRD that binds carbohydrate or glycolipid ligands in a calcium-dependent manner. Within the CRD, conserved calcium-binding residues and an EPN motif confer mannose-type ligand specificity.^96^ The transmembrane region harbors a positively charged arginine residue that mediates association with the ITAM-containing adapter Fc receptor γ chain (FcRγ) (Fig. 6). This interaction is essential for signal transduction. Upon ligand engagement, Mincle signals through the canonical FcRγ–Syk–CARD9–BCL10–MALT1 axis, culminating in NF-κB activation and induction of pro-inflammatory cytokines.^37^ Although Mincle employs a shared core signaling module, it acts as a context-dependent receptor rather than a binary switch. Pathogen-derived glycolipids and endogenous DAMPs engage the same proximal machinery but induce distinct transcriptional and functional outcomes depending on ligand affinity, receptor clustering, cellular context, and co-receptor or Toll-like receptor crosstalk, thereby fine-tuning cytokine responses instead of producing uniform outputs.^97,98^

In bone-resorbing cells, this signaling logic is mirrored by ITAM-dependent co-stimulatory receptors such as OSCAR and PIR-A, which also rely on FcRγ-mediated Syk activation. These receptors provide essential co-stimulatory signals that integrate with RANKL-driven pathways to promote osteoclast differentiation and function. Together, these findings highlight FcRγ–Syk signaling as a shared molecular platform through which immune receptors such as Mincle and osteoclast co-stimulatory receptors translate ligand diversity and environmental cues into distinct inflammatory and bone-destructive outcomes.^99,100^

Comparative analyses of bovine, murine, and human Mincle CRDs reveal a high degree of structural conservation in key ligand-binding residues, indicating preserved glycolipid recognition across species. Mutagenesis and crystallographic studies confirmed that the residues critical for trehalose and lipid interaction are conserved in mouse and human Mincle, suggesting functional parallels in receptor activation and downstream signaling.^101,102^

Mincle recognizes a broad spectrum of exogenous and endogenous ligands via its CRD. Its best-characterized ligands are the mycobacterial glycolipid trehalose dimycolate (TDM) and its synthetic analog trehalose-6,6′-dibehenate (TDB), which exhibit strong adjuvant activity and promote Th1 and Th17 responses.^37,95^ Mincle also binds microbial glycolipids from fungi and bacteria, supporting its role in antifungal and antibacterial immunity.^103^ In addition, Mincle detects endogenous danger-associated molecular patterns released during tissue damage, including SAP130, β-glucosylceramide, and cholesterol-derived ligands (Table 3).^10,104^

**Table 3:** Ligands for Mincle and their pathology

Ligands	Pathology	Reference
Trehalose dimycolate (TDM)	Tuberculosis (TB) infection, tissue inflammation and fibrosis	^134,135^
α-mannose	Atopic dermatitis, lethal sepsis	^136^
Sin3-associated protein 130 (SAP130)	Neuroinflammation in brain injury, kidney injury	^96,137^
β-glucosylceramide	Kidney injury	^138^

Through recognition of both microbial and self-derived signals, Mincle functions as a multifunctional sensor linking pathogen defense, sterile inflammation, and tissue homeostasis, while its potent adjuvant properties highlight its potential for vaccine development.

#### Mincle in bone remodeling

Accumulating evidence indicates that Mincle directly contributes to osteoimmune regulation and bone remodeling.^10,105^ Beyond its established role in innate immunity, Mincle shares structural and signaling features with classical osteoclast co-stimulatory receptors such as OSCAR and PIR-A, including dependence on FcRγ–Syk signaling. Importantly, experimental studies in murine models have demonstrated that Mincle activation directly influences osteoclast differentiation and bone resorptive activity, particularly under inflammatory conditions. The capacity of Mincle to sense necrosis-associated ligands, including SAP130 and endogenous lipid mediators such as β-glucosylceramide and cholesterol crystals, further links this receptor to sterile inflammatory environments characteristic of bone-destructive diseases such as rheumatoid arthritis and osteomyelitis.^105^ Together, these findings position Mincle as a context-dependent modulator of bone remodeling that integrates immune activation with osteoclast regulatory pathways, thereby contributing to inflammation-driven skeletal pathology.

### DC-SIGN (CD209)

DC-SIGN (dendritic cell-specific intercellular adhesion molecule-3-grabbing non-integrin, CD209) is a type II transmembrane C-type lectin receptor belonging to the group II CLR family. It is characterized by a calcium-dependent CRD that preferentially binds mannose- and fucose-rich glycans in a Ca²⁺-dependent manner. Unlike ITAM-bearing CLRs such as Dectin-1 or Mincle, DC-SIGN lacks a canonical signaling motif in its cytoplasmic tail but mediates immune signaling through interactions with adapter molecules and intracellular kinases. This signaling cascade leads to acetylation of the NF-κB subunit p65, resulting in transcriptional modulation of pro- and anti-inflammatory cytokines.^39,55^

DC-SIGN is predominantly expressed on myeloid dendritic cells (DCs), select macrophage subsets, and specialized tissue-resident myeloid populations, including dermal DCs and alveolar macrophages.^27,106^ Notably, dendritic cells, which often bear DC-SIGN, can differentiate into functional osteoclasts more efficiently than monocytes, particularly under stimulation with M-CSF and RANKL, as evidenced by genome-wide expression analyses showing DC-derived osteoclasts upregulate canonical osteoclast markers like cathepsin K and TRAP while downregulating DC-specific genes. This plasticity highlights a shared myeloid lineage commitment between DCs, macrophages, and osteoclasts.^107,108^ Its expression pattern reflects a dual function as both a pathogen recognition receptor and a key regulator of immune homeostasis. Under inflammatory conditions, DC-SIGN expression is further induced on monocyte-derived DCs, where it shapes cytokine production, antigen processing, and T cell priming.^109,110^ Through its CRD, DC-SIGN binds a broad range of high-mannose and fucose-containing glycans expressed by diverse pathogens, including *Mycobacterium tuberculosis*, *Helicobacter pylori*, and *Leishmania* species.^5,111,112^ These interactions facilitate pathogen uptake and antigen delivery for presentation, thereby influencing adaptive immune responses. In parallel, DC-SIGN recognizes endogenous glycoproteins such as ICAM-2 and ICAM-3, supporting immune cell adhesion, migration, and intercellular communication (Table 4).^113,114^ By recognizing both microbial and self-derived ligands, DC-SIGN acts as a context-dependent immunomodulatory hub.

**Table 4:** Ligands for DC-SIGN and their pathology

Ligands	Pathology	Reference
ICAM-2 and ICAM-3	Bacterial and viral infections, autoimmune disease	^139,140^
gp120	HIV infection	^141,142^
mannose and fucose-based oligosaccharides	SARS-CoV-2, human papillomavirus (HPV)	^143,144^
LPS	chronic inflammatory disease	^145,146^

Its signaling outcomes, shaped by ligand type and the cytokine environment, can promote protective immunity during infection or maintain tolerance in steady-state and chronic conditions. Dysregulation of DC-SIGN has been linked to persistent infections, chronic inflammation, and immune evasion, highlighting its central role in balancing immunity and tolerance.

#### DC-SIGN and osteoclastogenesis

DC-SIGN influences osteoclastogenesis indirectly through DC–osteoclast crosstalk and, under certain conditions, directly via DC-to-osteoclast trans-differentiation. While not expressed on mature osteoclasts, engagement of DC-SIGN on DCs activates NF-κB and Raf-1 signaling, driving production of pro-inflammatory cytokines such as TNF-α, IL-1β, and IL-6. These cytokines enhance RANKL expression in stromal cells, osteoblasts, and T cells, thereby promoting osteoclast precursor differentiation and bone resorption in inflammatory contexts, including rheumatoid arthritis and periodontitis.^5,27^ DC-SIGN^+^ immature DCs also retain myeloid plasticity and can trans-differentiate into osteoclasts upon RANKL/M-CSF or IL-17 stimulation, a process involving DC-STAMP–mediated fusion and contributing directly to inflammatory bone destruction, distinct from monocyte-derived osteoclasts.^8,115^ Importantly, DC-SIGN signaling exhibits ligand-dependent duality: pathogen-associated ligands, such as mycobacterial lipoarabinomannan, amplify pro-osteoclastogenic cytokines, whereas certain self-glycans induce IL-10, limiting excessive resorption.^116^ Together, these indirect and direct mechanisms position DC-SIGN as a context-dependent modulator of osteoclastogenesis, highlighting its potential as a target for therapeutic intervention in infection- or inflammation-driven bone loss.

## CLRs in bone pathology

CLRs, including Dectin-1, Mincle, and to a lesser extent DC-SIGN, integrate microbial and damage-associated signals in myeloid cells and play an important role in osteoclastogenesis. Beyond host defense, CLR-induced ITAM signaling provides essential co-stimulatory cues for osteoclast differentiation, as Syk-dependent calcium signaling synergizes with RANKL–RANK signaling to induce NFATc1.^4,43^ Consistent with this mechanism, increased expression of ITAM-associated molecules such as FcRγ has been observed in osteolytic lesions from rheumatoid arthritis, periodontitis, and periprosthetic tissues, linking chronic CLR-driven inflammation to pathological bone loss.^117^

CLR signaling converges on the Syk–CARD9–NF-κB axis and intersects with RANKL/ITAM co-stimulation to regulate both physiological bone remodeling and inflammatory bone destruction in diseases such as osteoporosis, rheumatoid arthritis, and periodontitis. In murine models of chronic rheumatoid arthritis, emergency myelopoiesis generates functionally reprogrammed myeloid cells. Notably, β-glucan–induced trained immunity skews myelopoiesis and osteoclast progenitors toward a heightened osteoclastogenic state, characterized by increased expression of pro-osteoclastogenic genes (e.g., Tnfsf11, Il6, Il1b, Il17a) upon secondary challenge. This trained state results in increased osteoclast formation and exacerbated bone loss in models of periodontitis and inflammatory arthritis, dependent on secondary inflammatory stimuli.^12,13,91^

### CLRs in inflammatory and degenerative bone diseases: rheumatoid arthritis (RA) and osteoporosis

While osteoporosis has traditionally been viewed as an endocrine disorder, it is now recognized as a condition with a strong inflammatory and osteoimmune component. Similarly, rheumatoid arthritis (RA) is an autoimmune disease characterized by chronic synovial inflammation, leading to cartilage degradation and focal bone erosion. Both conditions involve the activation and infiltration of myeloid cells and dysregulated osteoclastogenesis driven by inflammation. CLRs like Dectin-1 (CLEC7A), Mincle (CLEC4E), and DC-SIGN (CD209) are identified as key innate immune mediators linking chronic inflammation, tissue damage, and pathological bone remodeling. Acting through shared signaling modules such as Syk–CARD9–NF-κB and MAPK pathways, CLRs influence osteoclast differentiation, cytokine production, and synovial inflammation, thereby exacerbating or modulating bone and joint disease progression. CLR signaling synergizes with pro-inflammatory cytokines, such as TNF and IL-6, and with RANKL produced by synovial fibroblasts and T cells, thereby promoting osteoclast differentiation and localized bone resorption.^8,9^

In osteoporosis, especially in aging and postmenopausal settings, estrogen deficiency and metabolic stress promote the accumulation of damage-associated molecular patterns (DAMPs) such as oxidized lipids, necrotic debris, and microbiota-derived polysaccharides.^19,20,23^ These ligands activate Dectin-1 on bone marrow macrophages, enhancing NF-κB–dependent production of TNF-α, IL-1β, and IL-6 and increasing the sensitivity of osteoclast precursors to RANKL.^105^ Dectin-1 expression is elevated in ovariectomized mouse models and correlates with accelerated trabecular bone loss, while genetic or pharmacological inhibition of Dectin-1 or its downstream kinase Syk reduces osteoclast numbers and partially restores bone microarchitecture. Mincle further contributes by sensing endogenous ligands released during cell death, thereby sustaining low-grade inflammation and promoting osteoclastogenesis, whereas DC-SIGN appears to exert more regulatory effects by favoring IL-10 production and limiting excessive inflammatory cytokine release in certain contexts.^118^

In RA, the loss of immune tolerance towards self-antigens leads to T cell activation and cytokine production, recruiting other immune cells to the synovium.^68^ Chronic synovial inflammation results in continuous release of DAMPs from necrotic tissue and degraded cartilage, providing persistent stimuli for CLR signaling. Dectin-1 and Dectin-2 on synovial macrophages and dendritic cells promote, through Syk–ITAM signaling, the production of IL-6, IL-23, and other pro-inflammatory mediators that enhance TH17 responses and RANKL expression. Mincle expression is also markedly increased in RA synovium, particularly in CD14⁺ macrophages, and is further induced by TNF-α and IL-6. Preclinical studies demonstrate that Mincle deficiency attenuates osteoclast activation following osteocyte death, underscoring its potential contribution to RA-associated bone destruction. Nonetheless, the causative role of Mincle in RA pathogenesis, the identity of its synovial ligands, and the full spectrum of expressing cell types remain areas of active investigation.^10,21^ DC-SIGN is highly expressed by synovial macrophages in RA, with approximately 80% of CD68⁺ cells exhibiting receptor expression, in contrast to minimal expression in OA and trauma synovium. DC-SIGN⁺ macrophages are spatially associated with ICAM-3⁺ T cells and cartilage-degrading enzymes such as MMP-1 and EMMPRIN, suggesting a role in both immune cell retention and tissue destruction.^22^ Although regulatory T cells (Tregs) and anti-inflammatory cytokines such as IL-10 and IL-4 can inhibit osteoclastogenesis, persistent inflammation dominates, causing joint damage and deformation.

Collectively, CLRs such as Dectin-1, Mincle, and DC-SIGN constitute a critical interface between innate immune activation and bone-destructive pathways in RA. By sensing DAMPs in the inflamed synovium, these receptors integrate inflammatory cytokine signals and RANKL-driven osteoclastogenesis, thereby linking synovial inflammation to focal bone erosion. Understanding the precise roles, ligands, and signaling mechanisms of these CLRs may uncover novel therapeutic targets aimed at disrupting the innate immune–osteoclast axis in RA.

### C-type lectin receptors in periodontitis: linking microbial recognition to osteolysis

Periodontitis is a polymicrobial, dysbiosis-driven inflammatory disease in which chronic immune activation disrupts bone homeostasis and leads to osteoclast-mediated alveolar bone resorption. The shift toward an inflammatory microbiota enriches the biofilm with microbial ligands such as mannans, glycoproteins, and fungal β-glucans, which engage with PRRs on gingival immune cells and perpetuate destructive inflammation.^119,120^

Among these receptors, CLRs, including Dectin-1, Dectin-2, Mincle, and DC-SIGN, play a central role in recognizing carbohydrate structures and DAMPs. CLR expression on gingival macrophages and dendritic cells enables detection of dysbiotic microbial communities, thereby shaping both innate and adaptive immunity.^121^ Their engagement activates Syk-dependent and Raf-1–NF-κB signaling pathways, leading to robust production of pro-inflammatory cytokines, enhanced RANKL expression, and downstream activation of NFATc1, which synergizes with RANKL to promote osteoclastogenesis.^43^ Through these mechanisms, CLRs function as critical mediators linking pathogen recognition to inflammatory osteolysis. Toll-like receptor (TLR) pathways further amplify this response. Microbial components trigger TLR signaling in periodontal tissues, increasing pro-inflammatory cytokines and shifting the RANKL/OPG ratio toward sustained osteoclast differentiation. The combined activation of TLRs and CLRs establishes a feed-forward loop in which dysbiotic microbial communities continuously stimulate innate immune receptors, driving persistent inflammation and excessive osteoclastic bone resorption.^122^

Together, these findings position CLRs, alongside TLRs, as key regulators in the osteoimmune network of periodontitis, where their dysregulated activation by a carbohydrate-rich dysbiotic microbiota contributes to chronic inflammation, disruption of bone remodeling, and ultimately tooth loss.

Dectin-1 (CLEC7A) recognizes fungal β-glucans and contributes to antifungal defense. In periodontal tissues, Dectin-1 expression is upregulated in response to microbial challenge, including *Candida albicans*, and correlates with disease severity. Activation of Dectin-1 promotes Th1 responses, characterized by interferon-gamma (IFN-γ) production, enhancing pathogen clearance while also influencing local osteoclast activity through RANKL induction.^91^ Studies in murine pulpitis models have demonstrated that Dectin-1 expression correlates with TLR-2 and osteopontin (OPN), suggesting cooperative regulation of inflammatory and bone remodeling pathways.^123,124^

Mincle (CLEC4E) recognizes glycolipids and glycosylated proteins on pathogens, notably the S-layer of *Tannerella forsythia*, a key periodontal pathogen. The binding of Mincle to these ligands activates macrophages, leading to the secretion of pro-inflammatory cytokines such as TNF-α and IL-10. This response contributes to the inflammatory milieu, amplifying tissue destruction and promoting osteoclast recruitment.^125^

DC-SIGN (CD209), expressed on dendritic cells and macrophages, binds microbial carbohydrates including β-glucans, modulating cytokine production and T cell responses. In inflamed periodontal tissues, DC-SIGN expression has been linked to enhanced inflammatory signaling and potential osteoclastogenesis. Genetic polymorphisms in DC-SIGN may influence susceptibility to chronic periodontitis, suggesting a role in disease predisposition.

## Potential therapeutic implications

CLRs such as Dectin-1, Mincle, and DC-SIGN play central roles in antifungal immunity, inflammation, and bone metabolism, making them promising targets for treating osteoclast-mediated diseases. Among these, Dectin-1 and Mincle are well characterized for their regulatory effects on osteoclastogenesis. Activation of either receptor in osteoclast precursors suppresses differentiation through the Syk–NFATc1 pathway, suggesting therapeutic value in conditions marked by excessive bone resorption, including osteoporosis, rheumatoid arthritis (RA), and periodontitis.^10,15,105^

Dectin-1 signaling also activates the NLRP3 inflammasome and induces IL-1β, which can either promote inflammation and pathological osteoclast activity or suppress osteoclastogenesis under physiological conditions, highlighting its context-dependent effects.^126^ Reduced Dectin-1 expression in RA and systemic lupus erythematosus (SLE) correlates with higher disease activity, indicating that enhancing Dectin-1 signaling may help control autoimmune inflammation.^127^ Its cooperation with the inhibitory FcγRIIB receptor further amplifies its bone-protective and anti-inflammatory functions.^128^

Mincle recognizes glycolipids from pathogens and damage-associated molecular patterns from dying cells. Its activation triggers robust inflammatory responses and may drive inflammation-induced bone resorption. Thus, Mincle inhibition could reduce both inflammation and pathological bone loss in chronic inflammatory diseases.

**Fig. 7:**
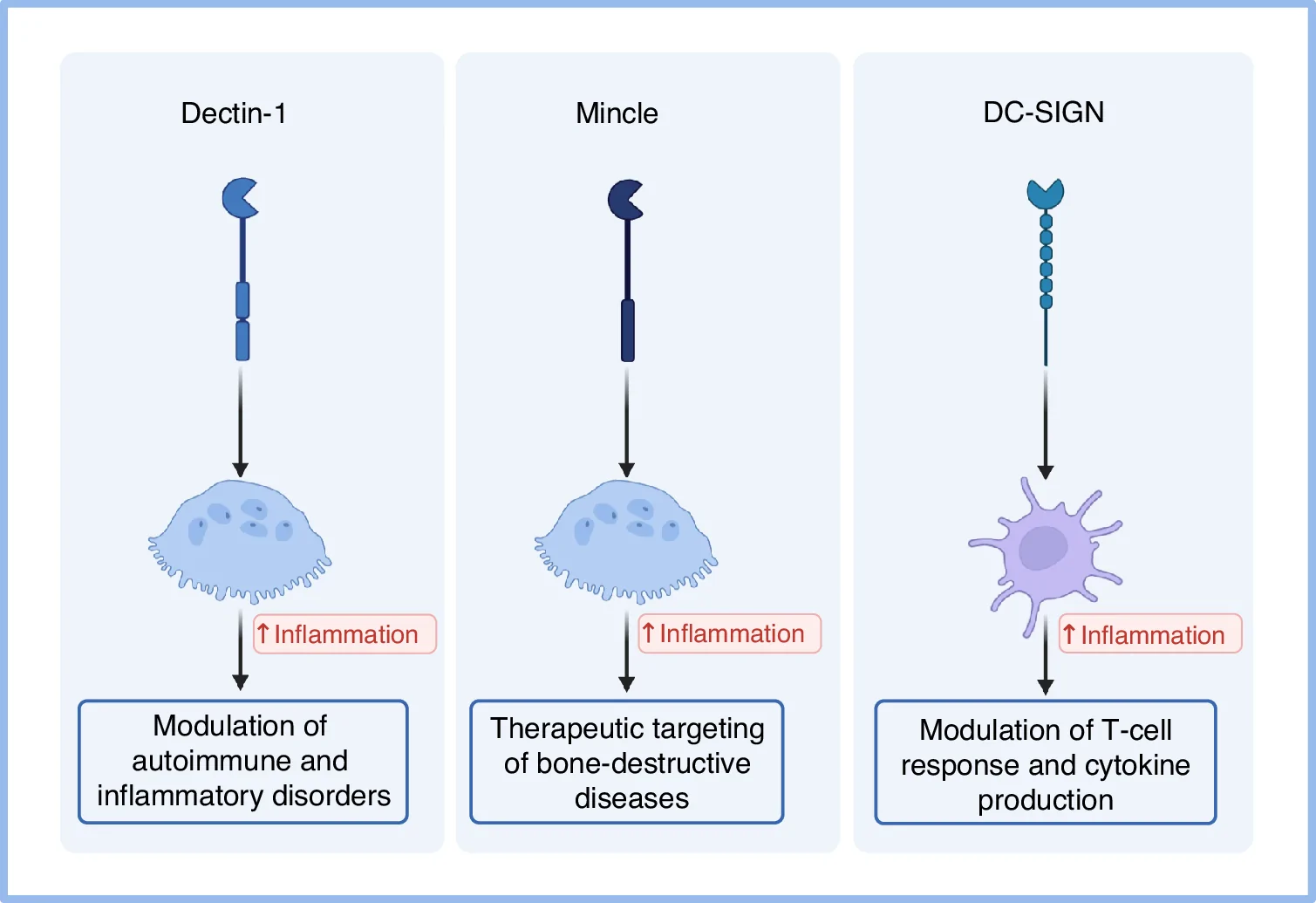
Therapeutic potential of CLR-targeted therapies. Targeting C-type lectin receptors (CLRs), including Dectin-1, Mincle, and DC-SIGN, may regulate osteoclastogenesis, inflammation, and immune responses. Dectin-1 and Mincle suppress osteoclast differentiation through Syk–NFATc1 signaling, while modulation of inflammatory pathways by Mincle and DC-SIGN may help prevent pathological bone loss in diseases such as osteoporosis, rheumatoid arthritis, and periodontitis

DC-SIGN, primarily expressed on dendritic cells, regulates cytokine production and T-cell responses through recognition of high-mannose glycans.^129^ Although it does not directly affect osteoclasts, its immune-modulatory effects can indirectly shape bone metabolism and complement the actions of Dectin-1 and Mincle (Fig. 7).

Compared with established anti-resorptive therapies such as bisphosphonates, SERMs, and denosumab, which effectively suppress osteoclast activity but are associated with systemic adverse effects, including osteonecrosis of the jaw, atypical femoral fractures, and hypocalcemia, CLR-targeted strategies may allow more selective modulation of osteoclastogenesis and inflammation. By integrating anti-resorptive and immune-regulatory functions, targeting CLRs such as Dectin-1, Mincle, and DC-SIGN represents a promising therapeutic avenue for osteoporosis, rheumatoid arthritis, periodontitis, and other inflammatory bone disorders, potentially through modulation of trained immunity in myeloid precursors. However, given the essential role of CLRs in antifungal and antibacterial host defense, therapeutic manipulation of these pathways may increase infection susceptibility, highlighting the need for careful safety evaluation and patient stratification (Fig. 7). Key challenges for translation include context-dependent signaling, targeted drug delivery to bone, and interactions with the oral microbiome. Tolerogenic ligands such as annexins may represent safer avenues for manipulating Dectin-1 and related CLRs.

## Future directions and conclusion

Future research on CLRs in bone biology should focus on resolving key knowledge gaps and exploiting emerging technologies to refine their therapeutic potential. Although Dectin-1, Mincle, and DC-SIGN are well characterized in osteoclast regulation, they are not expressed by osteoblasts or osteocytes, suggesting that their effects on bone formation and mechanotransduction are mediated indirectly through immune cell–derived signals. Defining how CLR activation in myeloid cells influences osteoblast differentiation, osteocyte function, and the regulation of RANKL and sclerostin would significantly advance current models of skeletal remodeling.

The cell type and context-specific functions of CLRs within the bone marrow niche, including roles in mesenchymal stem cells, macrophages, dendritic cells, and T cells, remain poorly understood, limiting therapeutic precision. Advanced approaches such as single-cell and spatial multi-omics, intravital imaging, and conditional genetic models will be essential to dissect CLR-dependent crosstalk between immune and skeletal cells in vivo. Notably, while murine studies suggest inhibitory roles for Dectin-1 and Mincle in osteoclast differentiation, the paucity of human data represents a major translational gap that warrants focused investigation, particularly regarding interactions with RANKL and co-stimulatory signaling pathways. Translating these findings into clinical applications holds strong potential for diseases such as RA, osteoporosis, osteomyelitis, and periodontitis.

Therapeutically, CLR-targeted strategies, including small-molecule modulators, monoclonal antibodies, engineered β-glucans, and biomaterial-based delivery systems, offer the potential for more selective control of osteoclast-driven inflammation and bone loss than conventional anti-resorptive or immunosuppressive therapies. However, given the central role of CLRs in antifungal and antibacterial immunity, achieving receptor specificity and cell-restricted modulation without compromising host defense remains a major challenge. Addressing ligand specificity, isoform-dependent signaling, developmental stage dependency, and genetic variability will be critical for advancing precision targeting of CLRs.

Overall, despite growing recognition of Dectin-1 and Mincle as regulators beyond host defense, their roles in human bone metabolism remain incompletely defined. Progress in this field will depend on integrated efforts across immunology, bone biology, and clinical research to translate mechanistic insights into safe and effective therapies for osteoclast-mediated bone diseases.
